# Different Land Use Intensities in Grassland Ecosystems Drive Ecology of Microbial Communities Involved in Nitrogen Turnover in Soil

**DOI:** 10.1371/journal.pone.0073536

**Published:** 2013-09-06

**Authors:** Annabel Meyer, Andreas Focks, Viviane Radl, Daniel Keil, Gerhard Welzl, Ingo Schöning, Steffen Boch, Sven Marhan, Ellen Kandeler, Michael Schloter

**Affiliations:** 1 Technische Universität München, Neuherberg, Germany; 2 Department for Aquatic Ecology and Water Quality Management, Wageningen, The Netherlands; 3 Helmholtz Zentrum München, German Research Centre for Environmental Health, Environmental Genomics, Neuherberg, Germany; 4 University of Hohenheim, Institute of Soil Science and Land Evaluation, Soil Biology Section, Stuttgart, Germany; 5 University of Jena, Institute of Ecology, Jena, Germany; 6 University of Bern, Institute of Plant Sciences, Bern, Switzerland; Wageningen University, The Netherlands

## Abstract

Understanding factors driving the ecology of N cycling microbial communities is of central importance for sustainable land use. In this study we report changes of abundance of denitrifiers, nitrifiers and nitrogen-fixing microorganisms (based on qPCR data for selected functional genes) in response to different land use intensity levels and the consequences for potential turnover rates. We investigated selected grassland sites being comparable with respect to soil type and climatic conditions, which have been continuously treated for many years as intensely used meadows (*IM*), intensely used mown pastures (*IP*) and extensively used pastures (*EP*), respectively. The obtained data were linked to above ground biodiversity pattern as well as water extractable fractions of nitrogen and carbon in soil. Shifts in land use intensity changed plant community composition from systems dominated by s-strategists in extensive managed grasslands to c-strategist dominated communities in intensive managed grasslands. Along the different types of land use intensity, the availability of inorganic nitrogen regulated the abundance of bacterial and archaeal ammonia oxidizers. In contrast, the amount of dissolved organic nitrogen determined the abundance of denitrifiers (*nirS* and *nirK*). The high abundance of *nifH* carrying bacteria at intensive managed sites gave evidence that the amounts of substrates as energy source outcompete the high availability of inorganic nitrogen in these sites. Overall, we revealed that abundance and function of microorganisms involved in key processes of inorganic N cycling (nitrification, denitrification and N fixation) might be independently regulated by different abiotic and biotic factors in response to land use intensity.

## Introduction

Soils provide a large number of ecosystem services including plant growth, carbon sequestration, degradation of xenobiotics and safeguarding of drinking water resources. Most of these functions are closely linked to the soil microbiome and its activity pattern [1], [2], [3]. Therefore many attempts were made to identify soil borne microbial communities as key drivers of ecological processes and describe factors that drive the abundance and diversity of selected functional communities [4]. Despite the high heterogeneity of soil microbes in time and space, it has become possible to figure out one general conclusions from these studies: Besides site-specific parameters, for example soil texture or climatic conditions, the type of land management and land use intensity has been identified as a major driver for microbial performance in soil [5], [6], [7], [8].

Recently, the effects of land use changes have been studied mainly focusing on (i) conversion of grassland to forest or vice versa [9], (ii) alterations in tillage management [10], [11], [12], (iii) changes in crop rotation [13] or (iv) modifications in fertilizer quality [14]. However, studies addressing questions related to consequences of changes in land use intensity on the soil microbiome are rare, although in many parts of the world we are facing a tremendous increase in land use intensity, due to the demands of bioeconomy (production of food, feed, fuel and fiber).

This intensification is also frequently observed in grassland ecosystems. While in the past sites have been used extensively as pastures, nowadays up to four times per season, the same areas are managed as meadows for hay production and silage, entailing an intensive application of organic and inorganic fertilizers. Differences in intensity of agricultural practice like mowing, grazing and fertilization lead to changes in plant composition [15], [16], [17], [13], microclimate, soil quality and hence to changes on macro- as well as micro-scale habitats. For some soil animals the impact of such changes is well known [18], [19], [20], [21] but data on microbial communities in soil is rare. For example, [6] compared diversity pattern of microbial community involved in nitrogen fixation, denitrification and nitrification in grassland ecosystems under different management intensities. This study clearly demonstrated changes in diversity pattern of single functional groups involved in nitrogen transformation on low diverse grassland sites. However, this study did not address questions how land use intensity influences the abundance and activity of selected functional groups of microbes in soil and thus changes turnover processes and rates.

The aim of the present study was to characterize microbial communities responsible for key processes in the inorganic nitrogen cycle (nitrification, denitrification and N-fixation) in grasslands of different land use intensity and relate these results to the aboveground biodiversity of plants as well as important below-ground properties (water extractable carbon and nitrogen fractions). Overall, we postulated that nitrogen cycling at extensively used sites is mainly driven by nitrogen fixation and internal nitrogen turnover is highly efficient resulting in low denitrification rates. In contrast, denitrification may play a more important role in nitrogen turnover at intensely used sites due to higher amounts of nitrogen available in soil and intensive plant growth (resulting in higher root exudation rates and increased microbial activity).

## Materials and Methods

### Experimental Setup and Sampling

Experiments were carried out in the frame of the German Biodiversity Exploratories [22], which form an ideal platform for such type of studies, as here for the first time gradients in land use intensity were defined on a large scale in three regions in Germany. For the present study soil samples was taken in 2008 in the southernmost Exploratory “Schwäbische Alb” which covers more than 45,000 ha of the state of Baden-Württemberg in SW-Germany. The mean annual precipitation in this area ranged from 938–963 mm, whereas the annual mean temperature was around 7°C. All sampled soils have been described as a Rendzic Leptosols with a clayey or loamy texture and a pH-value between 5.7 and 6.9. A more detailed soil description is given in table 1. Field work permits were given by the responsible state environmental offices of the state of Baden Württemberg (according to § 72 BbgNatSchG).

**Table 1:** Soil characteristics.

Site	Soil type^a^	Horizon^b^	Soil depth	Texture^c^			pH	Corg	N	C/N
				Sand	Silt	Clay				
			cm	– g kg^−1^ –				g kg^−1^	g kg^−1^	
IM 1^d^	Rendzic Leptosol	Ah	16	40	540	420	6.7	68.7	7.0	9.8
IM 2^d^	Rendzic Leptosol	Ah	19	140	650	220	6.9	41.5	4.7	8.8
IM 3^d^	Rendzic Leptosol	Ah	23	30	450	530	6.4	51.8	5.3	9.8
IP 1^e^	Vertic Leptosol	Ah	24	80	480	450	5.2	63.5	7.0	9.0
IP 2^e^	Rendzic Leptosol	Ah	21	60	690	250	6.4	83.6	8.5	9.8
IP 3^e^	Rendzic Leptosol	Ah	15	30	490	480	6.1	65.5	6.7	9.8
EP 1^f^	Rendzic Leptosol	Ah	14	280	530	190	7.2	40.4	3.3	12.2
EP 2^f^	Rendzic Leptosol	Ah	11	20	380	600	6.5	89.5	8.3	10.7
EP 3^f^	Rendzic Leptosol	Ah	27	40	680	270	6.7	67.6	5.9	11.5

a World Reference Base for soil resources, IUSS Working Group WRB.

b Horizon designation according to Guidelines for profile description, FAO.

c Soil texture was determined according to Schlichting & Blume (1966).

d intensely used meadow.

e intensely used mowed pasture.

f xtensively used pasture.

Nine different grassland sites (AEG1 - 9 with the given coordinates: N48° 23′ 56″ E9° 20′ 31″; N48° 23′ 15″ E9° 28′ 22″; N48° 24′ 29″ E9° 32′ 2″; N48° 23′ 38″ E9° 25′ 8″; N48° 23′ 48″ E9° 26′ 21″; N48° 24′ 11″ E9° 26′ 31″; N48° 23′ 33″ E9° 22′ 37″; N48° 26′ 13″ E9° 29′ 32″; N48° 23′ 44″ E9° 30′ 10″) were sampled categorized as follows: intensely used meadow (AEG 1–3 = *IM*, three times manure application and two times mown per season), intensely used mown pasture (AEG 4–6 = *IP*, grazed by cattle and horses, mown once a year and two times manure application per season) and extensively used pasture (AEG7 - 9 = *EP*, unfertilized but infrequently grazed by sheep). All sites were sampled in early spring (April) as well as in summer (July) to assess the effect of season.

From each site five sampling replicates were taken from the upper 10 cm; each replicate consist of five pooled bulk soil cores (d = 5.5 cm) taken with a soil auger. All samples were cooled directly after sampling for DNA based analyses at −20°C and at 4°C for measurements of enzyme activities and soil parameter.

### Plant Diversity

At all investigated sites the vegetation has been recorded on an area of 4 × 4 m. Plants were identified on taxa level and their percentage cover was estimated separately for the shrub layer (0–5 m woody species) and the herbaceous layer (including phanerophyte seedlings), respectively (data here not shown). The ecological strategy type of each vascular plant according to [23] were determined using the “Biolflor” data base (www.biolflor.de). This concept describing the general limits to ecology and evolution based on the trade-off that organisms face when the resources they gain from the environment are allocated between either growth, maintenance or regeneration – known as the universal three-way trade-off.: (C) the survival of the individual using traits that maximise resource acquisition and resource control in consistently productive niches, (S) individual survival via maintenance of metabolic performance in variable and unproductive niches, or (R) rapid gene propagation via rapid completion of the lifecycle and regeneration in niches where events are frequently lethal to the individual. Based on this assumption the csr triangle has been defined, in which each plant could be integrated according to its individual life type strategies. Furthermore, site conditions were characterized by calculating the mean indicator values based on plant species composition according to [24]. Ellenberg indicator values represent a well-established method for bio-indication of a range of environmental parameters.

### Chemical Analyses of Soils

CaCl_2_ extracts of the soil samples were prepared to analyze carbon and nitrogen contents in water extractable fraction. Therefore soil was mixed with 0.01 M CaCl_2_ at a ratio of 1∶2 [25] and homogenized by overhead shaken for 2 h followed by a filtration step. Water extractable organic carbon (WEOC) and total nitrogen bound (TNb) were measured using a DIMA-TOC 100 (DIMATEC Analysentechnik, Essen, Germany), nitrate and ammonium by using the commercial kits Nanocolor N°50 (NO_3_^–^N) and N°3 (NH_4_^+-^N) (Macherey-Nagel, Düren, Germany).

### Potential Nitrification and Denitrification Rate in Soils

Potential nitrification rates (PNR) were determined via micro titer plate assay as described by [26]. This method is based on the quantification of nitrite formed by ammonia oxidation. In brief, 2.5 g of fresh soil (three technical replicates for each soil sample), 50 µl sodium chlorate (1.5 M) and 10 ml ammonium sulfate (10 mM) were shaken for 5 h at 20°C whereas the added sodium chlorate should repress the oxidation of nitrite to nitrate. To stop the oxidation of ammonium 2.5 ml potassium chloride was added and incubated for 20 min. After short centrifugation 150 µl of the hydrous supernatant, 90 µl ammonium chloride (0.2 M) and 60 µl Griess-reagent (0.002 M naphthylenediamine dihydrochloride, 0.06 M sulphanilamide and 2.5 M phosphoric acid) were pipetted into the micro titer plates and the color change were detected via plate spectrometer at a absorbance wavelength of 540 nm (SpectraMax 340, MWG BIOTECH, Ebersberg, Germany). Nitrite concentrations of non-incubated soil samples served as control.

Potential denitrification activity (DEA) was determined using the anaerobic slurry technique as described by [27], slightly modified by [28]. Soil slurries, containing 10 g field-fresh soil and 10 ml of a mixture of 1 mM glucose/1 mM potassium nitrate (KNO_3_), were flushed with helium in airtight serum bottles. Subsequent acetylene was added to the slurries and the bottles were shaken via overhead shaker at 25°C for two hours. Every hour gas samples of headspace atmosphere were taken with a gas-tight syringe, and N_2_O concentrations measured using a gas chromatograph (GC-14B, Shimadzu, Japan).

### Nucleic Acid Extraction and Quantification

Genomic DNA was extracted from 0.5 g bulk soil (wet weight) using FastDNA Spin Kit for soil (MP Biomedicals, Germany) according to manufacturer’s protocol. Quality and Quantity of DNA extracts were determined with Nanodrop 1000 Spectrophotometer (Peqlab, Germany). A260/A280 ratios were approx 1.9. As the used kit is known to highly influence A230 due to the used binding matrix which has its absorption maximum at 230 nm, we used qPCR dilution series to exclude inhibition effects and did not calculate A230/260 ratios.

### Real-time PCR Assay

Quantitative real-time PCR (qPCR) was conducted on a 7300 Real-Time PCR System (Applied Biosystems, Germany) using SybrGreen as fluorescent dye to estimate the abundance of ammonia oxidizing bacteria and archaea, nitrogen fixing microbes as well as nitrite reducers. The marker genes used as well as the corresponding conditions for qPCR are listed in table 2.

**Table 2:** Thermal profiles and primer used for real-time PCR quantification of different functional genes.

Target gene	Source of standard	Thermal profile	No. ofcycles	Primer	Primer [µM]	DMSO[M]
*nifH*	*Shinorhiz. meliloti*	95°C-45 s/55°C–45 s/72°C-45s	40	nifHF [65] nifHR [65]	0.2	–
*amoA* AOA	Fosmid clone 54d9	94°C-45 s/55°C–45 s/72°C-45s	40	amo19F [40] CrenamoA616r48x [46]	0.2	–
*amoA* AOB	*Nitrosomonas* sp.	94°C-45 s/58°C–45 s/72°C-45s	40	amoA1F [66] amoA2R [66]	0.3	–
*nirK*	*Azospirillum irakense*	95°C-15 s/63–58°C-30 s/72°C-30s95°C-15 s/58°C–30 s/72°C-30 s	5^a^40	nirK876 [50] nirK5R [58]	0.2	0.3
*nirS*	*Pseudomonas stutzeri*	95°C-45 s/57°C–45 s/72°C-45 s	40	cd3aF [67] R3cd [68]	0.2	0.3

PCR reaction mixtures with a final volume of 25 µl consisted of Power SybrGreen Master Mix (12.5 µl), BSA (15 µg), template (2 µl) as well as primer and DMSO in a final concentration as referred in the table.

a Touchdown: −1°C cycle^−1.^

In a pre-experiment a dilution of the DNA of 1∶64 turned out to be best suited avoid inhibition of PCR, e.g. by co-extracted humic substances (data not shown). Quantitative real-time PCR was performed in 96–well plates (Applied Biosystems). Reaction mixtures with total volume of 25 µl were composed of 12.5 µl of Power SybrGreen PCR Master Mix (Applera, Germany), 15 µg bovine serum albumine (Sigma-Aldrich, Germany), gene specific concentrations of the forward and reverse primer (Metabion Germany) (see table 2) and 2 µl of diluted DNA extract. In the case of the genes *nirS* and *nirK* dimethyl sulfoxide (DMSO) was additionally used (final concentration of 0.3 M). The PCR reaction was started with a hot start of 94°C for 10 min according to the manufacturer’s instruction of the SybrGreen master mix, followed by 40 cycles with specific temperature profile according to the gene targeted (see table 2). Data collection was performed at each elongation step. The purity of the amplicons was checked by melting curve analysis and the presence of a unique band of the expected size in a 1.5% agarose gel stained with ethidium bromide. Standard curves was obtained using serial dilutions of plasmids DNA (10^6^–10^1^ gene copies/µl) containing the respective cloned gene (see table 2). The amplification efficiencies were calculated from the formula Eff = [10^(-1/slope)^ - 1] and resulted in values from 97% to 83%.

### Statistical Analysis

All results were related on the basis of one gram of soil dry weight (g dw^-1^). Using the R environment for statistical computing (http://www.R-project.org) univariate and multivariate methods were applied to test the effect of land use and season. Assumptions were tested by checking the residuals (equal variance in the groups, no outliers, normal distribution)**.** To account for the existence of replicate groups (n = 5) in each case linear mixed-effect models (lme in package nlme) were fitted. Two factor lmes with pairwise comparisons were used to construct a table of p-values. The integrative multivariate data analysis was based on a between group analysis (between in package ade4).

To investigate differences in plant diversity data a one factor ANOVA (lm in R) and subsequent pairwise t-tests with adjusted p-values (pairwise.t.test with method holm) were applied.

## Results

### Plant Diversity

The number of plant species on the investigated sites ranged between 17 and 58. Significantly lower numbers were observed on the intensively managed plots (24 respectively 25 for *IM* and *IP)* compared to the *EP* plots where 49 different species were found (p = 0.019). The coverage of vascular plant was between 102 and 170%, with highest plant coverage on the *IM* plots. The plant species composition showed a clear dependency on the land use intensity level (table S1). While grasses were dominant on the intensely used meadows (58 to 105% of coverage), the pastures (*EP* and *IP*) were more colonized by herbs (84 to 107% of coverage) indicating that grasses are more adapted to the disturbance caused by mowing. Considering the different ecological strategy types of the collected plants we found a clear trend for plants more linked to r-strategists on the extensively used sites (up to 96% of coverage) to c-strategists on the intensely used sites (up to 99% of the coverage).

The analysis of plants with indicator values for abiotic sites properties according to Ellenberg revealed a strong response of the plant community structure towards nutrient availability and soil water content (table 3). Most plant species from *EP* sites could be classified as indicators for nitrogen deficiency, whereas plants of intensely used sites were classified as indicators of moderate to strong nitrogen concentrations in soil. Additionally plants grown on the *EP* plots were better adapted to water shortage and matched well with plants being an indicator for moderate water shortage compared to plants obtained from the *IP* and *IM* plots which are indicators for moderate to well water conditions in soils.

**Table 3:** Soil parameters and plant analysis.

Site	WEOC^b^	Total WEN^c^	Nitrate	Ammonium	extracted DNA	Ellenberg indicator values for		Number and coversum of vascular plants	Number and cover sum of legumes
	April/July	April/July	April/July	April/July	April/July	N	moisture		
	µg C g^−1^	µg N g^−1^	µg N g^−1^	µg N g^−1^	µg DNA g^−1^			/[%]	/[%]
**IM** d **1 SD** a	53.3/49.0	32.4/23.3	34.6/18.2	0.7/0.2	6.0*10^4^/1.3*10^5^	4.9	4.4	26/111	4/20
	±3.8/±4.1	±8.4/±2.9	±12/±2.2	±0.2/±2.0*10^−2^	±1.1*10^4/^±1.3*10^4^				
**IM** d **2SD** a	39.2/56.7	50.8/23.0	44.7/17.2	0.6/19*10^−2^	4.7*10^4^/1.5*10^5^	6.6	5.4	17/126	1/8.0
	±4.5/±7.7	±9.4/±3.9	±7.7/±3.1	±0.2/±9.0*10^−2^	±1.2*10^3/^±3.3*10^4^				
**IM** d **3SD** a	33.0/37.2	15.3/9.38	17.4/8.05	3.0/26*10^−2^	3.9*10^4^/1.1*10^5^	5.7	5.4	28/171	3/31
	±5.7/±4.3	±7.2/±3.5	±8.7/±1.2	±0.2/±7.0*10^−2^	±5.7*10^3^/±1.7*10^4^				
**IP** e **1 SD** a	46.2/47.0	21.0/15.3	18.9/11.4	0.8/53*10^−2^	5.4*10^4^/1.0*10^5^	6.8	6.6	20/120	1/0.5
	±10/±4.8	±4.8/±3.8	±4.7/±3.1	±0.4/±15*10^−2^	±9.8*10^3^/±1.6*10^4^				
**IP** e **2 SD** a	57.4/51.1	62.6/17.8	54.5/12.7	1.4/25*10^−2^	5.9*10^4^/1.5*10^5^	6.4	5.9	20/102	1/0.5
	±7.0/±10	±20/±4.4	±18/±3.3	±0.6/±8.0*10^−2^	±9.7*10^3^/±1.7*10^4^				
**IP** e **3 SD** a	30.6/42.3	7.8/13.6	4.77/8.62	0.7/28*10^−2^	4.3*10^4^/1.2*10^5^	6.2	5.4	35/125	4/5.0
	±5.4/±5.5	±2.3/±2.0	±2.2/±1.5	±0.2/±8.0*10^−2^	±4.9*10^3^/±3.1*10^4^				
**EP** f **1 SD** a	43.5/31.1	6.9/4.1	1.88/1.10	1.6/28*10^−2^	4.0*10^4^/6.8*10^4^	2.7	3.5	46/106	4/3.0
	±2.6/±2.9	±2.9/±0.4	±1.0/±0.3	±0.9/±2.0*10^−2^	±8.4*10^3^/±8.7*10^3^				
**EP** f **2 SD** a	51.3/54.5	15.7/10.0	13.6/7.11	4.5/36*10^−2^	4.4*10^4^/3.6*10^4^	3.6	3.7	43/123	5/5.5
	±8.0/±5.2	±2.2/±2.1	±4.1/±1.7	±1.2/±3.0*10^−2^	±6.9*10^3^/±7.3*10^3^				
**EP** f **3 SD** a	42.7/44.5	42.7/44.5	1.61/1.94	0.8/23*10^−2^	3.5*10^4^/7.8*10^4^	2.9	3.5	58/119	6/5.5
	±4.7/±6.1	±2.4/±1.0	±1.3/±0.6	±0.3/±4.0*10^−2^	±6.6*10^3^/±7.6*10^3^				

a standard deviation.

b water-extractable organic carbon.

c water-extractable nitrogen.

d intensely used meadow.

e intensely used mowed pasture.

f xtensively used pasture.

### Labile Soil Nitrogen and Carbon Pools

Soil ammonium content ranged from 0.19 to 4.1 µg N g^−1^ dw and was up to ten times higher in spring than in summer (p = 0.0444). In July values were consistently low (0.19 and 0.53 µg N g^−1^ dw) in dependent from the site, whereas in April (0.62 to 4.1 µg N g^−1^ dw) differences between sites were more pronounced, with a tendency for higher values in soil samples from *EP* and *IM* compared to *IP* (p = 0.2976). Nitrate concentrations were in the range of 1.61 and 54.5 µg N g^−1^ dw in April and 1.10 and 18.2 µg N g^−1^ dw in July. No significant influence of land use and time was detected (p = 0.2162; p = 0.1246), however samples taken in April showed a tendency for higher values than those taken in July. Water extractable nitrogen (TNb) contents were strongly linked to the nitrate concentration with values from 4.85 to 62.6 µg N g^−1^ dw in April and in 4.09 to 23.3 µg N g^−1^ dw in July. WEOC values ranging from 30.6–57.4 µg C g^−1^ dw were neither affected by land use intensity (p = 0.9706) nor by season (p = 0.6837). Data are summarized in table 3 and 4.

**Table 4:** Statistical analysis.

Soil data	Ammonium	Nitrate		Total nitrogen	Organic carbon	Potential nitrification		Potential denitrification		DNA		*nirK*	*nirS*	*amoA* AOA	*amoA*AOB	*nifH*
Land use	0.5675	0.1246		0.1515	0.9706	0.0350*		0.0009*		0.0059		0.0048*	0.0498*	0.0064*	0.0004*	<0.0001*
Time point	0.0100*	0.2162		0.0993	0.6837	0.4882		0.2668		<0.0001*		<0.0001*	0.0315*	0.5872	0.0293*	0.656
Pairwise test																
*EP-IM*	n.s	n.s		n.s	n.s	0.0327*		0.0018*		0.0120*		0.0078*	0.0501*	0.0060*	0.0006*	0.0006*
*EP-IP*	n.s	n.s		n.s	n.s	0.2944		0.6746		0.0120*		0.0122*	0.2462	0.0530*	0.0014*	<0.0001*
*IM-IP*	n.s	n.s		n.s	n.s	0.2944		0.0540		0.8823		0.6737	0.2997	0.2116	0.5312	0.0030*
**Plant data**	**Plant** **diversity**	**Plant** **coverage**		**Legume** **diversity**	**Legume** **coverage**	**Ellenberg** **nitrogen**		**Ellenberg** **moisture**								
Land use	0.0110*		0.4200	0.0970	0.0400*		0.0009*		0.0027*							
Pairwise test																
*EP-IM*	0.0190*		n.s	n.s	0.7300		0.0029*		0.0184*							
*EP-IP*	0.0190*		n.s	n.s	0.6510		0.0012*		0.0028*							
*IM-IP*	0.8370		n.s	n.s	0.0590		0.1780		0.0640							

* significant at a level <0.05.

### Potential Nitrification and Denitrification Rates

**Figure 1:**
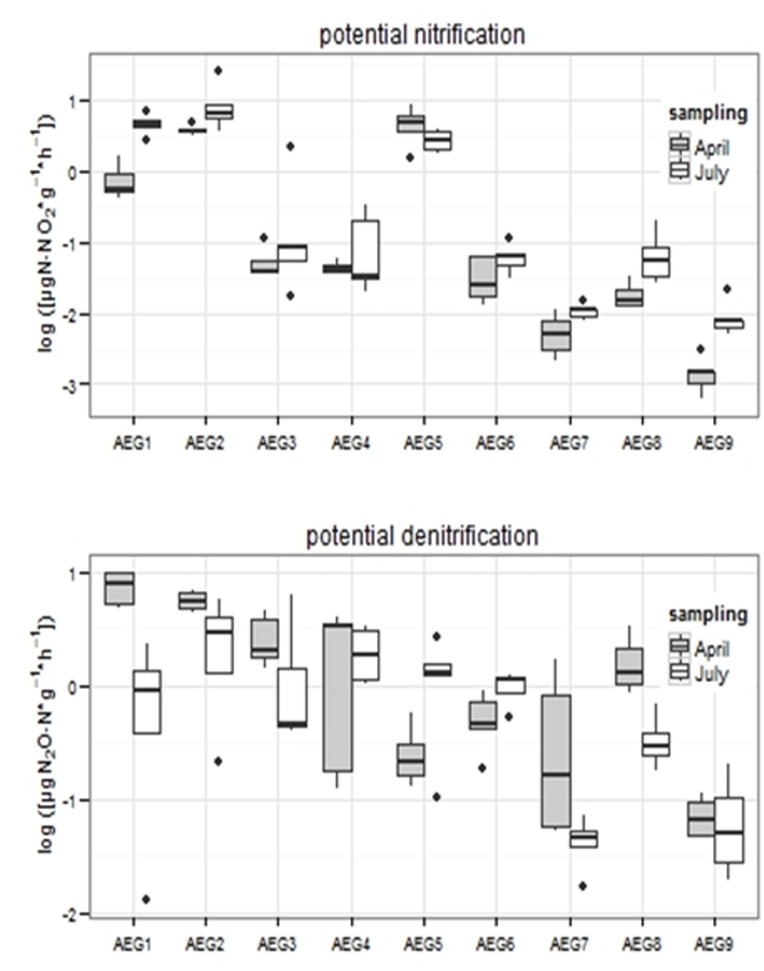
Box plot analysis of potential enzyme nitrification and denitrification activity at two different sampling time points (April and July) at 9 different grassland sites with different land use intensity. All data are log-transformed.

The highest PNR values were measured in samples derived from *IM* sites. However transformation rates between the three investigated sites (AEG1 - 3) were highly variable with values from 0.28 up to 2.55 µgNO_2_^−^ N g^−1^ dw h^−1^ (figure 1 and table 4). PNR in samples from plots characterized as *IP* ranged between 0.26 and 1.91 µgNO_2_^−^ N g^−1^ dw h^−1.^ and rates were neither significant different to *IM* nor to *EP* (p = 0.2944) plots. Again a high variation of the values, comparing the three sites under investigation (AEG4 - 6), was visible. The lowest PNR was measured in samples derived from *EP* (0.06−0.31 µgNO_2_^−^ N g^−1^ dw h^−1^), whereas a significant difference between *EP* and *IM* was observed (p = 0.0327). Overall a significant decrease of PNR from intensely to extensively managed sites was found (p = 0.0350). The sampling time point did not influence PNR in the investigated samples (p = 0.4882).

In contrast DEA showed an interdependency between the sampling time point and the land use intensity (p = 0.0346; figure 1 and table 4)). In spring the values for DEA in samples derived from *IM* and *EP* were up to the factor of three times higher (ranging from 0.31 to 2.39 µg N_2_O^−^ N g^−1^ dw h^−1^) compared to summer (0.25–1.46 µg N_2_O– N g^−1^ dw h^−1^) indicating a higher denitrification activity in soil at the beginning of the vegetation period. In samples from *IP* the contrary phenomenon was observed. Here higher values were measured in summer (0.99 to 1.34 µg N_2_O^−^ N g^−1^ dw h^−1^) than in spring (0.55 to 1.22 µg N_2_O^−^ N g^−1^ dw h^−1^). Overall a significant positive influence of land use intensity was proven (p = 0.0009).

### Abundances of Functional Genes

**Figure 2:**
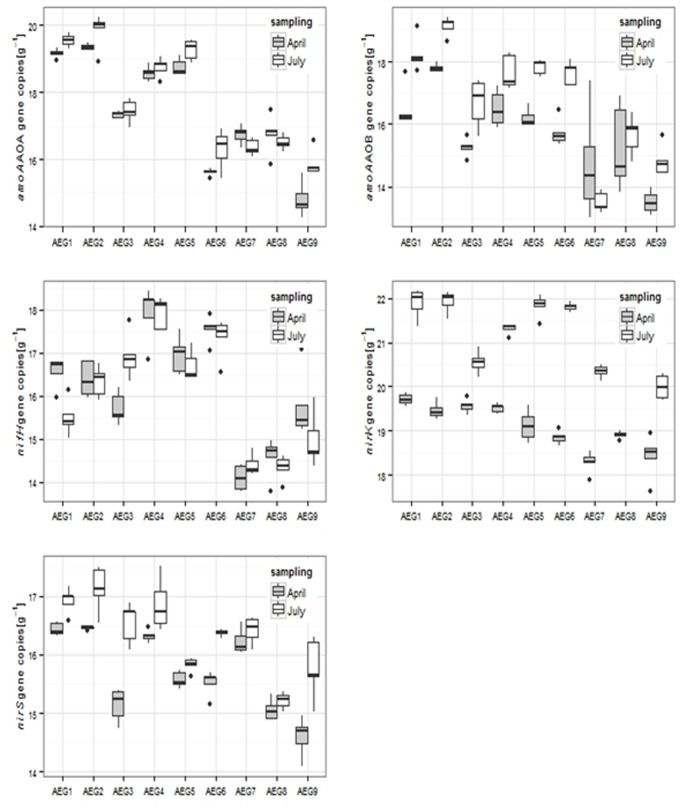
Box plot analysis of gene copy numbers of different genes involved in the cycling of inorganic nitrogen cycle at the two different sampling time points (April and July) with different land use intensity. All data are log-transformed.

Gene copy numbers of bacterial *amoA* genes (AOB) increased significantly from the spring to the summer sampling at all intensively managed sites (*IM* and *IP*), ranging in April from 4.4×10^6^ to 5.5×10^7^ copies g^-1^and in July from 2.2×10^7^ to 2.1 10^8^ copies g^-1^ (figure 2). Gene copy numbers in samples derived from the extensively used plots were in the range of 7.7×10^5^ and 8.7×10^6^ g^−1^ and hence significantly lower compared to the intensively managed sites (table 4). In addition, no clear seasonal effect could be described for samples derived from *EP* sites, as on two plots (AEG7 and AEG8) a decrease from April to July was observed whereas on the third one an increase was found. Overall, AOB community size was influenced by season (p = 0.0293) and by land use intensity (p = 0.0004). Gene copy numbers for the archaeal *amoA* gene (AOA) ranged from 3.0×10^6^ and 2.5×10^8 ^g^−1^ in April and from 8.5×10^6^ and 4.5×10^8^ copies g^−1^ in July; no statistically significant seasonal effect were proven (p = 0.5872), but a significant increase from intensely to extensively used plots was observed (see table 4). As for AOB also for AOA an increase from April to July of *amoA* gene abundances on the intensely used plots and a decrease on the extensively used sites was visible determined. Considering the ratios of AOA and AOB except the site AEG6 (AOA:AOB ratio <1) AOA : AOB ratios between 2.1 and 16 were found indicating a dominance from AOA over AOB. However in soil samples from the intensely used sites the ratio decreased significantly from April to July, whereas in samples from *EP* in two plots no changes were observed between the two sampling time points and a increase from 2.2 to 16 was found in the 3^rd^ plot (AEG7).

Gene copy numbers for *nirK* ranged in April from 8.9×10^7^ to 3.7×10^8^ copies g^−1^and increased in July ranging from 5.0×10^8^ to 3.4×10^9^ copies g^−1^ (p<0.0001; figure 2 and table 4). In April all plots of one land use category showed comparable abundance levels for *nirK*, whereas in July AEG3 (belonging to *IM*) revealed up to ten times lower copy numbers than AEG1 and 2 and AEG8 (belonging to *EP)* up to five times higher numbers than the other both plots of this land use category. Overall *nirK* gene abundance increased significantly with increasing land use intensity. The occurrence of *nirS* genes was significant lower in spring than in summer (p = 0.0315). Copy numbers ranged from 2.3×10^6^ and 1.4×10^7^ copies g^−1^ in April and between 4.0×10^6^ and 2.9×10^7^ copies g dw^-1^ in July. Also land use had a significant influence on the number of *nirS* gene copies. However *nirS* gene copy numbers were up to the factor of 500 lower compared to *nirK.*

Gene copy numbers for *nifH* ranged from 1.8×10^6^ to 6.9×10^7^ copies g dw^−1^ (figure 2). The data did not reveal significant differences between the sampling time points (p = 0.639) but between the land use intensities (p = 0.0006). *IP* sites show significant higher *nifH* copy numbers (from 1.9×10^7^ to 6.9×10^7^ copies g dw^−1^) than the other two land use categories. The lowest values were detected on the *EP* plots, where quantities between 1.4×10^6^ and 9.0×10^6^ copies g dw^−1^ were measured.

### Integrative Data Analysis

**Figure 3:**
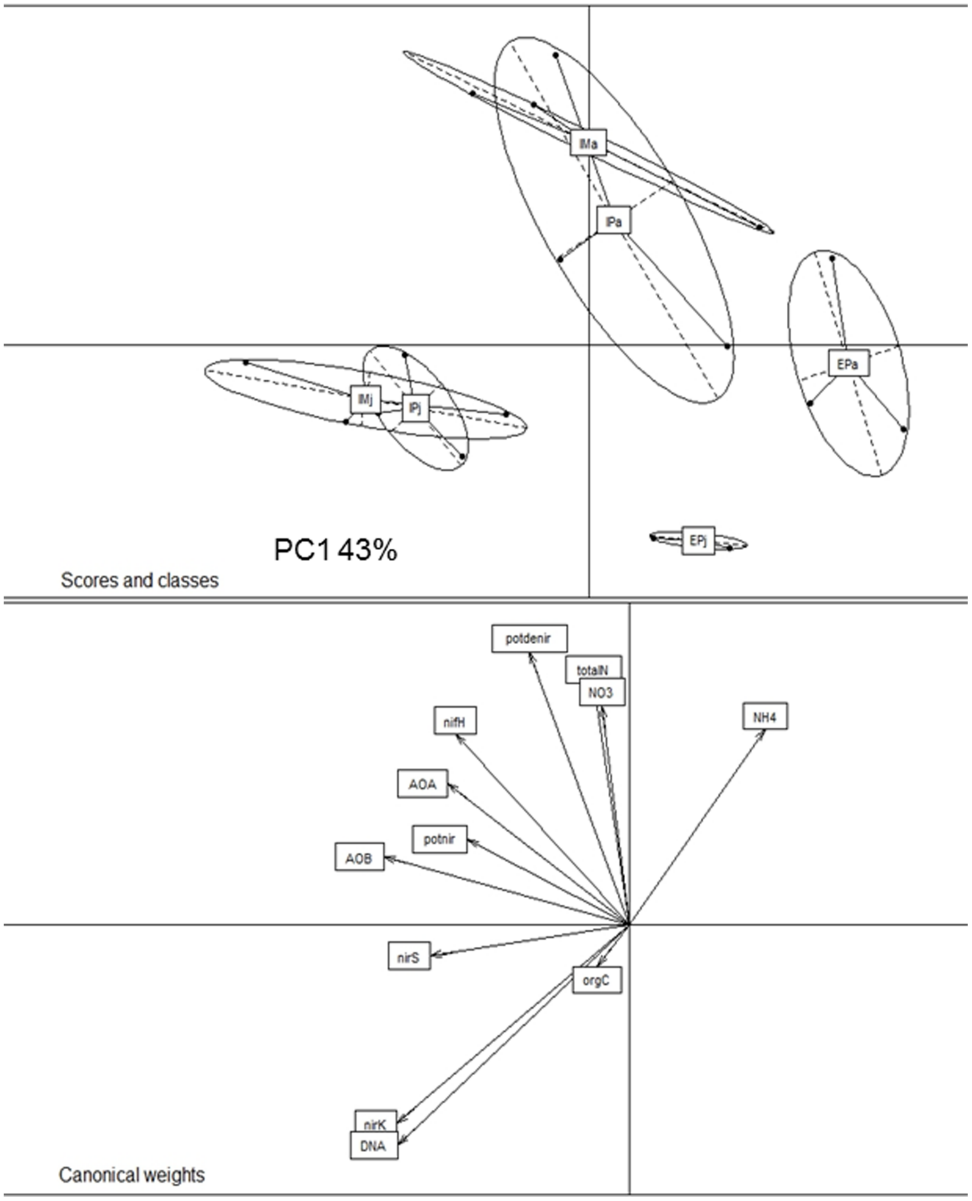
Between group analysis (BGA) using the mean values of all five replicates from each site. For calculation all data were logarithm-transformed, scaled and calculated using the R software package (www. r-project.org). The small letters a and j sign the April and July samples. Clear differentiation according to the sampling time point is visible (all April samples are on the right site in the upper part, all July on the left site in the lower part). Also the intensely used sites are clear separated from the extensively used plots whereas the separation is more clear in July.

Based on all data obtained a between group analysis (BGA) was performed using the mean values of all five replicates from each site (Figure 3). The BGA revealed fertilization and other types of soil disturbance (e.g. soil compaction by machines or animals) are interconnected and explain the differences between the intensely (*IM* and *IP*) and the extensively (*EP*) managed grassland sites to a large extent (PCA1∶43% PCA2∶24%). Based on the BGA data, it can be assumed that the effects of fertilization were more dominant compared to the influence of grazing and mowing. Moreover, clear differences between the sampling time points appeared (samples from July located in the lowerpart, from April in the upper part). Overall abundance of *nifH, amoA* AOA and AOB genes as well as potential nitrification activity were more related to land use intensity, whereas ammonium content and dissolved organic carbon as well as DNA content and abundance of *nirS* and *nirK* genes were affected more by time.

## Discussion

### Plant Diversity

[29] was among the first, who described a close link between land use intensity of grasslands and plant species composition. These observations have been confirmed in the present study. These results can be discussed on the basis of life strategies of plants and soil microorganisms: Whereas more plant species could be assigned to the exploitation group of so called c-strategists on the intensively managed sites, with high assimilation and growth rates if high nutrient levels were present in soil, the extensively managed plots were more characterized by “csr-plants” characterized by higher tolerance level to disturbance and nutrient stress but slower growth rates [23]. In addition to differences in species composition we found a significant reduction of plant species richness in response to land use intensification which has been also found in several other studies [30], [31], [32], [33]. These losses were comparable to an over regional inventory by [34] who revealed that increased fertilization by 90–130 kg N ha^−1^ is linked to a 50% higher productivity and a decrease of species richness by 30%.

### Land Use Intensity and Nitrification

Several studies which investigated the impacts of different management regimes on the nitrifying potential in soil [35], [6], [36] described higher PNR in response to higher intensities of land use of grassland ecosystems. These results were confirmed in our study where all intensely managed meadows showed higher PNR values compared to the extensively managed plots. Interestingly despite an increase in *amoA* gene copy numbers in samples from the *IP* and *IM* from spring to summer no comparable increase in PNR activities was observed. This could be the result of the fact that during summer the soil pH mainly in the rhizosphere decreased due to higher exudation rates of the plants, which might reduces the ratio of ammonia to ammonium in soil. As ammonia oxidizing microbes are only able to use ammonia for oxidation [37], this fact may explain that there is no direct link between PNR and *amoA* gene copies when data from the spring and summer sampling were compared. AOA harbor urease genes allowing AOA to use urea as alternative substrate, when ammonia is limited in soil [38]. The observed increase of AOA between spring and summer might be related to that urea utilization and a mixotrophic lifestyle of AOA. The relatively low PNR in summer (in relation to the abundance of AOA and AOB) might be also a fact of nitrification inhibitors which are excreted by many plants into soil to increase their competitiveness towards ammonia against microbes [39]. This ability of plants to reduce activity of AOA and AOB becomes even more important in those sites which have been extensively managed and nitrogen contents are very low. Obviously plants on these plots were indeed very competitive for ammonia uptake as not only PNR rates did not change between spring and summer but also the abundance of AOA and AOB did not change.

In nearly all samples a higher number of *amoA* AOA than *amoA* AOB copies (AOA/AOB ratios were between 2 and 16) was found. These results are in accordance with some other recent studies indicating dominance from AOA over AOB in soils [40], [41], [14]. Up to now it is still unclear if these higher abundance values of AOA might be related to a significant contribution to ammonification in soils [42], [43], [44], [45], [46]. However considering the correlation of different *amoA* genes and the nitrification potential (R_(AOA)_ = 0.836, p_(AOA)_ <0.001 and R_(AOB)_ = 0.680, p_(AOB )_<0.001) our dataset delivers one more hint that AOA plays an important role at least for ammonia oxidation in soil, although based on molecular data still the oxidation of hydroxylamine to nitrite has not been proven for AOA. Overall it seems that AOA and AOB are similarly affected by land use intensification and it has been crystallized that nitrogen availability is the major driver for performance and abundance of the nitrifying community.

### Land Use Intensity and Denitrification

Microbial communities in soil are generally stimulated by plant growth especially in the rhizosphere by root exudates, resulting in an increase of anoxic habitats in the rhizosphere and the need for microbes to use alternative electron acceptors like nitrate inducing denitrification [47], [48]. This was confirmed in our study by reduced DEA activities in soil samples derived from *EP*, where plant biomass was significantly lower. However in our study DEA was not stimulated in summer compared to spring, which is surprising on the first glance but might be a result of a low nitrate concentrations found mainly in soils from intensively managed plots *IP* and *IM*. Furthermore the differences in water content and the corresponding differences in the redox potential between spring and summer sampling might have influenced DEA activity independent from land use intensities.

To describe denitrifying communities, the two nitrite reductase genes (*nirK* and *nirS*) were quantified. Overall, many prokaryotes from soil are able to denitrify and the proportion of denitrifiers within the soil microbial community was considered between 10% and 60% of the total bacterial and archaeal microbiota reaching values between 10^5^ and 10^9^ copies g^−1^ [27], [49], [50], [51], [52], [53]. The values found in our study were in a similar range. In all samples gene copy numbers for *nirK* genes dominated over *nirS* genes *(nirS/nirK* ratio 0.002–0.02). This is in accordance with data from other studies, which indicate *nirK* phylotypes being more dominant in the rhizosphere or in soils characterized by an intensive root development [54], or high nutrient contents due to climatic conditions (e.g. freezing and thawing, [55]). The relatively high WEOC (water extractable organic carbon) values confirmed this hypothesis (Table 3). A *s*trong time effect (p<0.0001) was found for both types of nitrite reductase genes in all soil samples, with values up to 20 times higher in summer, which correlated to the increased overall microbial biomass values obtained in soil samples from summer compared to spring (data not shown). The increase in abundance of the nitrite reductase over time is in contrast to the potential measurements of denitrification, but could be easily explained as all denitrifiers are facultative anaerobes and can shift their metabolism from respiration to denitrification based on the conditions present in soil. Therefore not surprisingly this missing link between denitrifying activity and genetic potential was described in some studies before [56], [57].

However several studies have indicated that the plant species composition can act as a major driver for denitrifiers in soil [58] probably by providing different amounts and compositions of root exudates [59], [60] mainly in summer which may explain the significant copy number increase of both genes in our study. Overall it can be concluded that abundance of denitrifiers is mainly driven by the amount and quality of carbon present in soil and consequently increase or decrease of denitrifiers is strongly linked to development of overall microbial biomass, whereas the induction of genes involved in denitrification is linked to the presence of nitrate and the redox conditions present in soil.

### Land Use Intensity and Nitrogen Fixation

Surprisingly the highest amount of *nifH* copies was found at *IP* sites and not as we assumed at sites, which have been used extensively (*EP*) were nitrogen is limited. Despite no differences were found in the WEOC, this might be related to the fact that in soil the amount of energy equivalents needed for nitrogen fixation is too low in soil samples derived from *EP* [*61*], confirming earlier data obtained by [62] from glacier fore fields, where also low abundance of *nifH* as well as low potential activity for nitrogen fixation was found at the youngest sites, which had the lowest supply with nitrogen and lacked of energy equivalents. [63] as well as [64] showed in their study that intensive grazing has positive influences on nitrogen fixing communities in grassland soil, due to the high amounts of excrements which provide good sources for energy generation.

However it must be stated that the occurrence of legumes was not correlated to the *nifH* gene abundance, so we assume that the obtained results just reflect the occurrence of free living nitrogen fixing microbes, as we did not include nodule analysis in our study.

### Conclusion

Obviously there is a strong link between land use intensity and microbial community structure in soil. Some of these effects might be direct effects and a response to e.g. increased fertilization regimes, other might be indirect effects and mediated by changes in the plant diversity and biomass (e.g. through changes in exudation). However the described data just reflect potentials for certain processes. If the same response pattern can be confirmed for the activation of certain functional traits remains an open question for future research focusing more on mRNA based studies with a more dense net of sampling time points.

## Supporting Information

Table S1 List of plant species with species categorization according to ecological strategy type (Grime 1997) and Ellenberg values for moisture (M) and nutrients (N) (Ellenberg et. al. 2001). Different ecological strategy types as given in the table are competitors (c), competitive ruderals (cr), stress-tolerant competitors (cs), stress-tolerant ruderals (sr), csr-plants (competition is restricted by the combined effects of stress and disturbance), stress-tolerants (s) and ruderals (r). Ellenberg indicator values are normalized values on an ordinal scale from low to high values (1 to 9) whereas 1 means high tand 9 low tolerance to nutrient- and moisture stress. NA shows plants which are not categoriesed by Ellenberg and x signs these with a large ecolocial amplitude. Occurence of each plant species on the different plots is given in percent of coverage.
(DOC)

## References

[1] Singh DK (2008) Biodegradation and bioremediation of pesticide in soil: concept, method and recent developments. Indian Journal of Microbiology 48: 35–40. 23100698 10.1007/s12088-008-0004-7 PMC3450205

[2] Lillis L, Doyle E, Clipson N (2009) Comparison of DNA- and RNA-based bacterial community structures in soil exposed to 2,4-dichlorophenol. Journal of Applied Microbiology 107: 1883–1893. 20426769 10.1111/j.1365-2672.2009.04369.x

[3] Sinha S, Chattopadhyay P, Pan I, Chatterjee S, Chanda P, et al (2009) Microbial transformation of xenobiotics for environmental bioremediation. African Journal of Biotechnology 8: 6016–6027.

[4] Ollivier J, Töwe S, Bannert A, Hai B, Kastl EM, et al (2011) Nitrogen turnover in soil and global change. FEMS Microbiology Ecology 78: 3–16. 21707675 10.1111/j.1574-6941.2011.01165.x

[5] Dilly O, Blume HP, Sehy U, Jimenez M, Munch JC (2003) Variation of stabilized, microbial and biologically active carbon and nitrogen in soil under contrasting land use and agricultural management practices. Chemosphere 52: 557–569. 12738293 10.1016/S0045-6535(03)00237-6

[6] Patra AK, Abbadie L, Clays-Josserand A, Degrange V, Grayston SJ, et al (2006) Effects of management regime and plant species on the enzyme activity and genetic structure of N-fixing, denitrifying and nitrifying bacterial communities in grassland soils. Environmental Microbiology 8: 1005–1016. 16689721 10.1111/j.1462-2920.2006.00992.x

[7] Cookson WR, Osman M, Marschner P, Abaye DA, Clark I, et al (2007) Controls on soil nitrogen cycling and microbial community composition across land use and incubation temperature. Soil Biology and Biochemistry 39: 744–756.

[8] Robson TM, Lavorel S, Clement JC, Le Roux X (2007) Neglect of mowing and manuring leads to slower nitrogen cycling in subalpine grasslands. Soil Biology & Biochemistry 39: 930–941.

[9] Berthrong ST, Schadt CW, Pineiro G, Jackson RB (2009) Afforestation alters the Composition of Functional Genes in Soil and Biogeochemical Processes in South American Grasslands. Applied and Environmental Microbiology 75: 6240–6248. 19700539 10.1128/AEM.01126-09 PMC2753075

[10] Cheneby D, Brauman A, Rabary B, Philippot L (2009) Differential responses of nitrate reducer community size, structure, and activity to tillage systems. Applied and Environmental Microbiology 75: 3180–3186. 19304827 10.1128/AEM.02338-08 PMC2681619

[11] Vargas Gil S, Meriles J, Conforto C, Figoni G, Basanta M, et al (2009) Field assessment of soil biological and chemical quality in response to crop management practices. World Journal of Microbiology and Biotechnology 25: 439–448.

[12] Attard E, Poly F, Commeaux C, Laurent F, Terada A, et al (2010) Shifts between *Nitrospira*- and *Nitrobacter*-like nitrite oxidizers underlie the response of soil potential nitrite oxidation to changes in tillage practices. Environmental Microbiology 12: 315–326. 19807778 10.1111/j.1462-2920.2009.02070.x

[13] Kaschuk G, Alberton O, Hungria M (2009) Three decades of soil microbial biomass studies in Brazilian ecosystems: Lessons learned about soil quality and indications for improving sustainability. Soil Biology & Biochemistry 42: 1–13.

[14] Hai B, Diallo NH, Sall S, Haesler F, Schauss K, et al (2009) Quantification of key genes steering the microbial nitrogen cycle in the rhizosphere of sorghum cultivars in tropical agro-ecosystems. Applied and Environmental Microbiology 75: 1034–1043. 10.1128/AEM.02917-08 PMC2725491 19502431

[15] Thurston JM (1969) The effect of liming and fertilizers on the botanical composition of permanent grassland and the yield of hay. (Thurston JM ed.), 3–10. Blackwell, Oxford

[16] Kirkham FW, Mountford JO, Wilkins RJ (1996) The effects of nitrogen, potassium and phosphorus addition on the vegetation of a Somerset peat moor under cutting management. Journal of Applied Ecology 33: 1013–1029.

[17] Wedin DA, Tilman D (1996) Influence of nitrogen loading and species composition on the carbon balance of grasslands. Science 274: 1720–1723. 8939865 10.1126/science.274.5293.1720

[18] O’Neill KM, Olson BE, Wallander R, Rolston MG, Seibert CE (2010) Effects of Livestock Grazing on Grasshopper Abundance on a Native Rangeland in Montana. Environmental Entomology 39: 775–786. 20550790 10.1603/EN09173

[19] Mills A, Adl MS (2006) The effects of land use intensification on soil biodiversity in the pasture. Canadian Journal of Plant Science 86: 1339–1343.

[20] Parfitt RL, Yeates GW, Ross DJ, Schon NL, Mackay AD, et al (2010) Effect of fertilizer, herbicide and grazing management of pastures on plant and soil communities. Applied Soil Ecology 45: 175–186.

[21] Postma-Blaauw MB, de Goede RGM, Bloem J, Faber JH, Brussaard L (2010) Soil biota community structure and abundance under agricultural intensification and extensification. Ecology 91: 460–473. 20392011 10.1890/09-0666.1

[22] Fischer M, Bossdorf O, Gockel S, et al (2010) Implementing large-scale and long-term functional biodiversity research: The Biodiversity Exploratories. Basic and Applied Ecology 11: 473–485.

[23] Grime JP (1977) Evidence for the existence of three primary strategies in plants and its relevance to ecological and evolutionary theory. The American Naturalist 111: 1169–1194.

[24] Ellenberg H., Weber H.E., Düll R., Wirth V. (2001) Zeigerwerte von Pflanzen in Mitteleuropa. Institute for Phytogeography, University of Göttingen, Göttingen

[25] Houba V, Novozamsky I, Huybregts A, van der Lee J (1986) Comparison of soil extractions by 0.01 M CaCl_2_, by EUF and by some conventional procedures. Plant and Soil 96: 433–437.

[26] Hoffmann H, Schloter M, Wilke BM (2007) Gradients of potential microbial nitrification rates in soil aggregates; Biology and Fertility of Soils. 44: 411–413.

[27] Tiedje JM (1988) Ecology of denitrification and dissimilatory nitrate reduction to ammonium. John Wiley and Sons, New York

[28] Šimek M, Hopkins DW (1999) Regulation of potential denitrification by soil pH in long-term fertilized arable soils. Biology and Fertility of Soils 30: 41–4.

[29] Tilman (1987) Secondary succession of pattern of plants dominate experimental nitrogen gradients. Ecological Monographies 57: 1898–1914.

[30] Scott D, Shay J (1990) Competition, Fire, and Nutients in a Mixed-Grass Prairie. Ecology 71: 1959–1967.

[31] Jacquemyn H, Brys R, Hermy M (2003) Short-term effects of different management regimes on the response of calcareous grassland vegetation to increased nitrogen. Biological Conservation 111: 137–147.

[32] Maurer K, Weyand A, Fischer M, et al (2006) Old cultural traditions, in addition to land use and topography, are shaping plant diversity of grasslands in the Alps.Biological Conservation. 110: 438–446.

[33] Klimek S, Richter G, Kemmermann A, Hofmann M, Isselstein J (2007) Plant species richness and composition in managed grasslands: the relative importance of field management and environmental factors. Biological. Conservation 134: 559–570.

[34] Gough L, Osenberg CW, Gross C, Collins S (2000) Fertilization effects on species density and primary productivity in herbaceous plant communities. Oikos 89: 428–439.

[35] Le Roux X, Bardy M, Loiseau P, Louault F (2003) Stimulation of soil nitrification and denitrification by grazing in grasslands: do changes in plant species composition matter? Oecologia 137: 417–425. 12955489 10.1007/s00442-003-1367-4

[36] Le Roux X, Poly F, Currey P, et al (2008) Effects of aboveground grazing on coupling among nitrifier activity, abundance and community structure. ISME Journal 2: 221–232. 18049458 10.1038/ismej.2007.109

[37] Gubry-Rangin C, Hai B, Quince C, Engel M, Thomson BC, et al (2011) Niche specialization of terrestrial archaeal ammonia oxidizers; PNAS. 108: 21206–21211. 10.1073/pnas.1109000108 PMC3248517 22158986

[38] Tourna M, Stieglmeier M, Spang A, Koenneke M, Schintlmeister A, et al. (2011) *Nitrosophaera vinnensis*, an ammonia oxidizing archaeaon from soil; PNAS 108, 8420–8425. 10.1073/pnas.1013488108 PMC3100973 21525411

[39] Subbarao GV, Nakahara K, Hurtado MP, Ono H, Morata DE, et al (2009) Evidence for biological nitrification inhibition in Brachiaria pastures. Proceedings of the National Academy of Sciences (USA) 106: 7302–1730. 10.1073/pnas.0903694106 PMC2752401 19805171

[40] Leininger S, Urich T, Schloter M, et al (2006) Archaea predominate among ammonia-oxidizing prokaryotes in soils. Nature 442: 806–809. 16915287 10.1038/nature04983

[41] Babic KH, Schauss K, Hai B, Sikora S, Redzepovic S, et al (2008) Influence of different *Sinorhizobium meliloti* inocula on abundance of genes involved in nitrogen transformations in the rhizosphere of alfalfa (*Medicago sativa* L.). Environmental Microbiology 10: 2922–2930. 18973619 10.1111/j.1462-2920.2008.01762.x

[42] Di HJ, Cameron KC, Shen JP, He JZ, Winefield CS (2009) A lysimeter study of nitrate leaching from grazed grassland as affected by a nitrification inhibitor, dicyandiamide, and relationships with ammonia oxidizing bacteria and archaea. Soil Use and Management 25: 454–461.

[43] Jia ZJ, Conrad R (2009) Bacteria rather than Archaea dominate microbial ammonia oxidation in an agricultural soil. Environmental Microbiology 11: 1658–1671. 19236445 10.1111/j.1462-2920.2009.01891.x

[44] Mertens J, Broos K, Wakelin SA, Kowalchuk GA, Springael D, et al (2009) Bacteria, not archaea, restore nitrification in a zinc-contaminated soil. ISME Journal 3: 916–923. 19387487 10.1038/ismej.2009.39

[45] Offre P, Prosser JI, Nicol GW (2009) Growth of ammonia-oxidizing archaea in soil microcosms is inhibited by acetylene. FEMS Microbiology Ecology 70: 99–108. 19656195 10.1111/j.1574-6941.2009.00725.x

[46] Schauss K, Focks A, Leininger S, Kotzerke A, Heuer H, et al (2009) Dynamics and functional relevance of ammonia-oxidizing archaea in two agricultural soils. Environmental Microbiology 11: 446–456. 19196275 10.1111/j.1462-2920.2008.01783.x

[47] Drew MC, Lynch JM (1980) Soil anaerobiosis, microorganisms, and root function. Annual Review of Phytopathology 18.

[48] Zausig JR, Stepniewski W, Horn R (1993) Oxygen Concentration and redox potential Gradients in Unsaturated Model Soil Aggregates. Soil Science Society of America Journal 57: 908–916.

[49] Vinther FP, Eiland F, Lind AM, Elsgaard L (1999) Microbial biomass and numbers of denitrifiers related to macropore channels in agricultural and forest soils. Soil Biology and Biochemistry 31: 603–611.

[50] Henry S, Baudoin E, Lopez-Gutierrez JC, Martin-Laurent F, Brauman A, et al (2004) Quantification of denitrifying bacteria in soils by nirK gene targeted real-time PCR. Journal of Microbiological Methods 59: 327–335. 15488276 10.1016/j.mimet.2004.07.002

[51] Henry S, Bru D, Stres B, Hallet S, Philippot L (2006) Quantitative detection of the *nosZ* gene, encoding nitrous oxide reductase, and comparison of the abundances of 16S rRNA, *narG*, *nirK*, and *nosZ* genes in soils. Applied and Environmental Microbiology 72: 5181–5189. 16885263 10.1128/AEM.00231-06 PMC1538733

[52] Dandie CE, Burton DL, Zebarth BJ, Henderson SL, Trevors JT, et al (2008) Changes in Bacterial Denitrifier Community Abundance over Time in an Agricultural Field and Their Relationship with Denitrification Activity. Applied and Environmental Microbiology 74: 5997–6005. 18689522 10.1128/AEM.00441-08 PMC2565952

[53] Hallin S, Jones CM, Schloter M, Philippot L (2009) Relationship between N-cycling communities and ecosystem functioning in a 50-year-old fertilization experiment. ISME Journal 3: 597–605. 19148144 10.1038/ismej.2008.128

[54] Sharma S, Aneja MK, Mayer J, Munch JC, Schloter M (2005) Diversity of transcripts of nitrite reductase genes (*nirK* and *nirS*) in rhizospheres of grain legumes. Applied and Environmental Microbiology 71: 2001–2007. 15812032 10.1128/AEM.71.4.2001-2007.2005 PMC1082563

[55] Sharma S, Szele Z, Schilling R, Munch JC, Schloter M (2006) Influence of freeze-thaw stress on the structure and function of microbial communities and denitrifying populations in Soil. Applied and Environmental Microbiology 72: 2148–2154. 16517665 10.1128/AEM.72.3.2148-2154.2006 PMC1393215

[56] Enwall K, Philippot L, Hallin S (2005) Activity and composition of the denitrifying bacterial community respond differently to long-term fertilization. Applied and Environmental Microbiology 71: 8335–8343. 16332820 10.1128/AEM.71.12.8335-8343.2005 PMC1317341

[57] Philippot L, Hallin S (2005) Finding the missing link between diversity and activity using denitrifying bacteria as a model functional community. Current Opinion in Microbiology 8: 234–239. 15939345 10.1016/j.mib.2005.04.003

[58] Bremer C, Braker G, Matthies D, Reuter A, Engels C, et al (2007) Impact of plant functional group, plant species, and sampling time on the composition of *nirK*-type denitrifier communities in soil. Applied and Environmental Microbiology 73: 6876–6884. 17766442 10.1128/AEM.01536-07 PMC2074960

[59] Mounier E, Hallet S, Chèneby D, et al (2004) Influence of maize mucilage on the diversity and activity of the denitrifying community. Environmental Microbiology 6: 301–312. 14871213 10.1111/j.1462-2920.2004.00571.x

[60] Henry S, Texier S, Hallet S, et al (2008) Disentangling the rhizosphere effect on nitrate reducers and denitrifiers: insight into the role of root exudates. Environmental Microbiology 10: 3082–3092. 18393993 10.1111/j.1462-2920.2008.01599.x

[61] Myrold DD, Bottomley PJ (2007) Biological N inputs. (Paul EA, eds.), 365–388. Elsivier, Burlington

[62] Brankatsch R, Töwe S, Kleineidam K, Zeyer J, Schloter M (2011) Abundances and potential activities of nitrogen cycling microbial communities along a glacier chronosequence; The ISME Journal. 5: 1025–1037. 10.1038/ismej.2010.184 PMC3131848 21124490

[63] Hou F, Nan Z, Xiao J, Chang S (2002) [Characteristics of vegetation, soil, and their coupling of degraded grasslands]. Ying Yong Sheng Tai Xue Bao 13: 915–922. 12418247

[64] Patra AK, Abbadie L, Clays-Josserand A, et al (2005) Effects of grazing on microbial functional groups involved in soil N dynamics. Ecological Monographs 75: 65–80.

[65] Rösch C, Mergel A, Bothe H (2002) Biodiversity of denitrifying and dinitrogen-fixing bacteria in an acid forest soil. Applied Environmental Microbiology. 68: 3818–3829. 10.1128/AEM.68.8.3818-3829.2002 PMC124007 12147477

[66] Rotthauwe JH, Witzel KP, Liesack W (1997) The ammonia monooxygenase structural gene *amoA* as a functional marker: molecular fine-scale analysis of natural ammonia-oxidizing populations. Applied Environmental Microbiology 63: 4704–4712. 9406389 10.1128/aem.63.12.4704-4712.1997 PMC168793

[67] Michotey V, Méjean V, Bonin P (2000) Comparison of methods for quantification of cytochrome *cd* _1_-denitrifying bacteria in environmental marine samples. Applied Environmental Microbiology 66: 1564–1571. 10742243 10.1128/aem.66.4.1564-1571.2000 PMC92024

[68] Throbäck IN, Enwall K, Jarvis A, Hallin S (2004) Reassessing PCR primers targeting *nirS*, *nirK* and *nosZ* genes for community surveys of denitifying bacteria with DGGE. FEMS Microbiology Ecology 49: 401–417. 19712290 10.1016/j.femsec.2004.04.011

