# Supplementary material for: Different Land Use Intensities in Grassland Ecosystems Drive Ecology of Microbial Communities Involved in Nitrogen Turnover in Soil
Source: PLoS One. 2013 Sep 6;8(9):e73536. doi: 10.1371/journal.pone.0073536 (PMC3765351; doi:10.1371/journal.pone.0073536)
Supplement: Table S1 — List of plant species with species categorization according to ecological strategy type (Grime 1997) and Ellenberg values for moisture (M) and nutrients (N) (Ellenberg et. al. 2001). Different ecological strategy types as given in the table are competitors (c), competitive ruderals (cr), stress-tolerant competitors (cs), stress-tolerant ruderals (sr), csr-plants (competition is restricted by the combined effects of stress and disturbance), stress-tolerants (s) and ruderals (r). Ellenberg indicator values are normalized values on an ordinal scale from low to high values (1 to 9) whereas 1 means high tand 9 low tolerance to nutrient- and moisture stress. NA shows plants which are not categoriesed by Ellenberg and x signs these with a large ecolocial amplitude. Occurence of each plant species on the different plots is given in percent of coverage. (DOC) [file pone.0073536.s001.doc]

Table S1: List of plant species with species categorization according to ecological strategy type (Grime 1997) and Ellenberg values for moisture (M) and nutrients (N) (Ellenberg et. al. 2001). Different ecological strategy types as given in the table are competitors (c), competitive ruderals (cr), stress-tolerant competitors (cs), stress-tolerant ruderals (sr), csr-plants (competition is restricted by the combined effects of stress and disturbance), stress-tolerants (s) and ruderals (r). Ellenberg indicator values are normalized values on an ordinal scale from low to high values (1 to 9) whereas 1 means high tand 9 low tolerance to nutrient- and moisture stress. NA shows plants which are not categoriesed by Ellenberg and x signs these with a large ecolocial amplitude. Occurence of each plant species on the different plots is given in percent of coverage.

| Plant species | Ecological strategy | Ellenberg indicator | | *IM* | | | *IP* | | | *EP* | | |
| --- | --- | --- | --- | --- | --- | --- | --- | --- | --- | --- | --- | --- |
|  |  | M | N | AEG1 | AEG2 | AEG3 | AEG4 | AEG5 | AEG6 | AEG7 | AEG8 | AEG9 |
| *Polygala amarella* | csr | 9 | 1 | . | . | . | . | . | . | 0.5 | . | 0.5 |
| *Teucrium montanum* | csr | 1 | 1 | . | . | . | . | . | . | 1 | . | . |
| *Thymus pulegioides ssp. carniolicus* | csr | 2 | 1 | . | . | . | . | . | . | 10 | 0.5 | 3 |
| *Thymus pulegioides ssp. pulegioides* | csr | 4 | 1 | . | . | . | . | . | . | 1 | 3 | 1 |
| *Anthyllis vulneraria* | csr | 3 | 2 | . | . | . | . | . | . | 0.5 | . | . |
| *Briza media* | csr | x | 2 | . | . | . | . | . | . | 5 | 1 | 4 |
| *Campanula rotundifolia* | csr | x | 2 | . | . | . | . | . | . | 1 | 0.5 | 0.5 |
| *Carex caryophyllea* | csr | 4 | 2 | . | . | . | . | . | . | 1 | 0.5 | 2 |
| *Carlina acaulis ssp. simplex* | csr | 4 | 2 | . | . | . | . | . | . | 0.5 | . | 0.5 |
| *Cirsium acaule* | csr | 3 | 2 | . | . | . | . | . | . | 3 | . | 2 |
| *Galium pumilum* | csr | 4 | 2 | . | . | . | . | . | . | 0.5 | . | 0.5 |
| *Gentiana verna* | csr | 4 | 2 | . | . | . | . | . | . | . | . | 0.5 |
| *Helianthemum nummularium ssp. obscurum* | cs | 3 | 2 | . | . | . | . | . | . | . | . | 7 |
| *Helictotrichon pratense* | cs | 3 | 2 | 25 | . | 10 | . | . | . | 1 | 0.5 | 0.5 |
| *Hieracium pilosella* | csr | 4 | 2 | . | . | . | . | . | . | 1 | 0.5 | 0.5 |
| *Hippocrepis comosa* | csr | 3 | 2 | . | . | . | . | . | . | . | 0.5 | . |
| *Koeleria macrantha* | cs | 3 | 2 | . | . | . | . | . | . | 1 | . | 0.5 |
| *Koeleria pyramidata ssp. pyramidata* | cs | 4 | 2 | . | . | . | . | . | . | 1 | 0.5 | 2 |
| *Linum catharticum* | sr | x | 2 | . | . | . | . | . | . | 0.5 | . | 0.5 |
| *Ononis repens ssp. procurrens* | cs | 4 | 2 | . | . | . | . | . | . | 1 | . | . |
| *Pimpinella saxifraga* | cs | 3 | 2 | . | . | . | . | . | . | 0.5 | 0.5 | 0.5 |
| *Polygala comosa* | csr | 3 | 2 | . | . | . | . | . | . | . | . | 0.5 |
| *Polygala vulgaris* | csr | 4 | 2 | . | . | . | . | . | . | 0.5 | . | . |
| *Potentilla heptaphylla* | csr | 3 | 2 | . | . | . | . | . | . | 1 | . | 0.5 |
| *Potentilla tabernaemontani* | csr | 3 | 2 | . | . | . | . | . | . | 0.5 | . | 2 |
| *Sanguisorba minor* | csr | 3 | 2 | . | . | . | . | . | . | 5 | 2 | 2 |
| *Veronica teucrium* | c | 3 | 2 | . | . | . | . | . | . | 0.5 | 3 | . |
| *Asperula cynanchica* | csr | 3 | 3 | . | . | . | . | . | . | . | . | 0.5 |
| *Bromus erectus* | cs | 3 | 3 | . | . | . | . | . | . | 4 | 40 | 25 |
| *Bromus hordeaceus* | cr | x | 3 | 1 | 2 | . | 2 | . | 1 | . | . | . |
| *Carex montana* | csr | 4 | 3 | . | . | . | . | . | . | 1 | . | . |
| *Euphorbia cyparissias* | csr | 3 | 3 | . | . | . | . | . | . | 7 | 3 | 2 |
| *Galium verum* | cs | 4 | 3 | . | . | . | . | . | . | 3 | 2 | 0.5 |
| *Leucanthemum ircutianum* | c | 4 | 3 | 0.5 | . | . | . | . | . | . | . | . |
| *Leucanthemum vulgare* | c | 4 | 3 | . | . | . | . | . | . | . | 0.5 | 0.5 |
| *Lotus corniculatus* | csr | 4 | 3 | . | . | . | . | . | . | 1 | 0.5 | 0.5 |
| *Luzula campestris* | csr | 4 | 3 | . | . | . | . | . | . | . | 1 | 0.5 |
| *Origanum vulgare ssp. vulgare* | csr | 3 | 3 | . | . | . | . | . | . | . | 0.5 | . |
| *Plantago media* | csr | 4 | 3 | . | . | 2 | . | . | . | 1 | 2 | 0.5 |
| *Poa angustifolia* | cs | x | 3 | . | . | . | . | . | 0.5 | . | 15 | 1 |
| *Prunella grandiflora* | csr | 3 | 3 | . | . | . | . | . | . | 10 | . | 6 |
| *Ranunculus bulbosus ssp. bulbosus* | csr | 3 | 3 | . | 2 | . | . | . | 0.5 | 3 | 2 | 2 |
| *Rhinanthus alectorolophus* | csr | 4 | 3 | . | . | . | . | . | 1 | . | . | . |
| *Rhinanthus minor* | csr | 4 | 3 | . | . | . | . | . | . | 0.5 | . | 2 |
| *Rosa rubiginosa* | c | 3 | 3 | . | . | . | . | . | . | . | . | 0.5 |
| *Scabiosa columbaria* | csr | 3 | 3 | . | . | . | . | . | . | 0.5 | 0.5 | 2 |
| *Trifolium campestre* | r | 4 | 3 | . | . | . | . | . | . | . | . | 0.5 |
| *Trifolium medium* | c | 4 | 3 | . | . | . | . | . | . | . | . | 0.5 |
| *Agrimonia eupatoria ssp. eupatoria* | **c** | 4 | 4 | . | . | . | . | . | . | . | 0.5 | 0.5 |
| *Alchemilla monticola* | csr | 5 | 4 | . | . | . | 0.5 | . | 0.5 | . | . | . |
| *Brachypodium pinnatum* | csr | 4 | 4 | . | . | . | . | . | . | 12 | . | 15 |
| *Carex flacca* | csr | 6 | 4 | . | . | . | . | . | . | 3 | . | 1 |
| *Cerastium arvense ssp. arvense* | cr | 4 | 4 | . | . | . | . | . | . | . | 0.5 | . |
| *Crataegus monogyna* | c | 4 | 4 | . | . | . | . | . | . | . | . | 0.5 |
| *Cynosurus cristatus* | csr | 5 | 4 | . | . | . | . | . | 0.5 | . | 4 | 0.5 |
| *Daucus carota ssp. carota* | cr | 4 | 4 | . | . | . | . | . | . | . | . | 0.5 |
| *Euphrasia officinalis ssp. rostkoviana* | r | x | 4 | . | . | . | . | . | . | 1 | . | 0.5 |
| *Helictotrichon pubescens ssp. pubescens* | c | 3 | 4 | . | . | . | . | . | . | . | 3 | 0.5 |
| *Hypericum perforatum* | c | 4 | 4 | . | . | . | . | . | . | . | . | 0.5 |
| *Trifolium dubium* | r | 4 | 4 | . | . | . | . | . | 0.5 | . | . | . |
| *Achillea millefolium* | c | 4 | 5 | . | 1 | 0.5 | . | . | . | 2 | 8 | 0.5 |
| *Bistorta officinalis* | csr | 7 | 5 | . | . | . | 2 | . | . | . | . | . |
| *Cerastium holosteoides* | cr | 5 | 5 | 0.5 | 3 | 0.5 | 0.5 | 1 | 0.5 | . | 0.5 | . |
| *Crepis biennis* | cr | 6 | 5 | 0.5 | . | 0.5 | . | 0.5 | 4 | . | . | . |
| *Galium mollugo agg.* | c | 4 | 5 | 8 | . | 5 | . | 5 | 4 | . | 0.5 | . |
| *Leontodon autumnalis ssp. autumnalis* | csr | 5 | 5 | . | . | . | . | . | . | . | . | 0.5 |
| *Trisetum flavescens* | csr | x | 5 | 25 | 15 | 50 | 1 | 25 | 25 | . | 2 | . |
| *Vicia sepium* | c | 5 | 5 | 5 | . | 1 | . | 0.5 | 2 | . | . | . |
| *Bellis perennis* | csr | 5 | 6 | 0.5 | 0.5 | 0.5 | . | . | . | . | . | . |
| *Carum carvi* | c | 5 | 6 | 1 | 10 | . | . | . | 0.5 | . | . | . |
| *Dactylis glomerata ssp. glomerata* | c | 5 | 6 | 5 | 8 | 2 | 2 | 1 | 12 | . | 3 | . |
| *Festuca pratensis ssp. pratensis* | c | 6 | 6 | . | . | 0.5 | 10 | . | 2 | . | 5 | . |
| *Leontodon hispidus* | csr | 5 | 6 | . | . | . | . | . | . | 0.5 | . | 0.5 |
| *Myosotis arvensis ssp. arvensis* | r | 5 | 6 | 0.5 | 0.5 | . | 0.5 | 0.5 | 0.5 | . | . | . |
| *Poa pratensis* | c | 5 | 6 | . | 8 | 0.5 | 0.5 | 1 | 1 | . | . | . |
| *Rumex acetosa* | c | x | 6 | 1 | . | 1 | 0.5 | 10 | . | . | . | . |
| *Tragopogon pratensis ssp. orientalis* | csr | 5 | 6 | . | . | . | . | . | 0.5 | . | . | . |
| *Tragopogon pratensis ssp. pratensis* | csr | 4 | 6 | . | . | . | . | . | 0.5 | . | . | . |
| *Trifolium repens* | csr | 5 | 6 | 10 | 8 | 20 | . | . | 0.5 | 1 | 3 | 0.5 |
| *Alopecurus pratensis* | c | 6 | 7 | . | . | . | 5 | 20 | 15 | . | . | . |
| *Arrhenatherum elatius* | c | x | 7 | . | 15 | . | . | 0.5 | 30 | . | . | . |
| *Geranium sylvaticum* | c | 6 | 7 | . | . | 3 | . | . | . | . | . | . |
| *Glechoma hederacea* | csr | 6 | 7 | . | . | 0.5 | . | . | . | . | . | . |
| *Lolium perenne* | c | 5 | 7 | 1 | . | 10 | 10 | 5 | 2 | . | . | . |
| *Phleum pratense* | c | 5 | 7 | . | . | . | . | . | 0.5 | . | . | . |
| *Poa trivialis ssp. trivialis* | csr | 7 | 7 | 1 | 20 | 30 | 45 | 20 | 2 | . | . | . |
| *Ranunculus repens* | csr | 7 | 7 | 0.5 | . | . | 35 | . | . | . | . | . |
| *Anthriscus sylvestris ssp. sylvestris* | c | 5 | 8 | 10 | . | . | 0.5 | 2 | 2 | . | . | . |
| *Geranium pyrenaicum* | csr | 5 | 8 | . | 1 | . | . | . | . | . | . | . |
| *Heracleum sphondylium* | c | 5 | 8 | . | . | 5 | 0.5 | 5 | 3 | . | . | . |
| *Silene dioica* | c | 6 | 8 | 0.5 | . | . | . | . | . | . | . | . |
| *Taraxacum sect. ruderalia* | csr | 5 | 8 | 5 | 30 | 2 | 2 | 2 | 3 | . | 4 | . |
| *Lamium album ssp. album* | csr | 5 | 9 | . | . | . | . | 2 | 2 | . | . | . |
| *Rumex obtusifolius* | c | 6 | 9 | . | 1 | . | 2 | . | . | . | . | . |
| *Alchemilla vulgaris agg.* |  | NA | NA | . | . | 0.5 | . | . | . | . | . | . |
| *Hieracium subg. pilosella* |  | NA | NA | . | . | . | . | . | . | . | . | 0.5 |
| *Tragopogon pratensis* | csr | NA | NA | 0.5 | . | . | . | . | . | . | . | . |
| *X festulolium loliaceum* |  | NA | NA | . | . | . | . | . | 0.5 | . | . | . |
| *Anthoxanthum odoratum* | csr | x | x | . | . | 2 | . | . | . | . | 2 | 3 |
| *Arenaria serpyllifolia* | r | 4 | x | . | . | . | . | . | . | 0.5 | . | . |
| *Colchicum autumnale* | csr | 6 | x | . | . | 0.5 | . | . | . | . | . | . |
| *Festuca guestfalica* |  | 4 | x | . | . | . | . | . | . | 15 | 3 | 12 |
| *Festuca rubra ssp. rubra* | c | 6 | x | . | . | . | . | . | . | . | 1 | 0.5 |
| *Juniperus communis ssp. communis^S* | c | 4 | x | . | . | . | . | . | . | 1 | . | . |
| *Medicago lupulina* | csr | 4 | x | 0.5 | . | . | . | . | . | 0.5 | 0.5 | 0.5 |
| *Plantago lanceolata* | csr | x | x | 0.5 | 1 | 10 | . | . | 0.5 | 0.5 | 0.5 | 2 |
| *Prunella vulgaris* | csr | 5 | x | . | . | 0.5 | . | . | . | 0.5 | 0.5 | 0.5 |
| *Ranunculus acris* | c | 6 | x | 2 | . | 3 | . | 0.5 | 3 | . | . | . |
| *Trifolium pratense ssp. pratense* | c | 5 | x | 5 | . | 10 | 0.5 | . | 2 | . | 1 | 3 |
| *Veronica arvensis* | r | x | x | 1 | . | . | 0.5 | 0.5 | 1 | . | . | . |
| *Veronica chamaedrys* | csr | 5 | x | . | . | 0.5 | . | 0.5 | 1 | 0.5 | 1 | . |
| *Glyceria_fluitans* | cs | NA | NA | . | . | . | . | . | . | . | 0.5 | 0.5 |
